# Validation of living with chronic illness scale in a type 2 diabetes mellitus population

**DOI:** 10.1186/s12955-021-01715-x

**Published:** 2021-03-17

**Authors:** Jorge Caro-Bautista, Carmen Rodríguez-Blázquez, David Perez-Manchon, Eva Timonet-Andreu, Gloria Carvajal-Carrascal, Alejandra Fuentes-Ramírez, Silvia Corchon, Marta Aranda-Gallardo, Leire Ambrosio

**Affiliations:** 1 Andalusian Public Health System, District of Primary Health Care of Málaga-Valle del Guadalhorce and Instituto de Investigación Biomédica de Málaga-IBIMA, Málaga, Spain; 2 National Centre of Epidemiology and CIBERNED, Carlos III Institute of Health, Madrid, Spain; 3 Faculty of Health, Camilo José Cela University, Madrid, Spain; 4 Department of Cardiology, Costa del Sol Hospital and Instituto de Investigación Biomédica de Málaga-IBIMA, Málaga, Spain; 5 Facultad de Enfermería y Rehabilitación, Universidad de La Sabana, Chía, Colombia; 6 Faculty of Nursing and Chiropody, University of Valencia, Valencia, Spain; 7 Department of Internal Medicine, Costa del Sol Hospital, Marbella, Málaga Spain; 8 School of Health Sciences, NIHR ARC Wessex, University of Southampton, Building 67, University Road, SO171BJ Southampton, United Kingdom

**Keywords:** Long-term condition, Type 2 diabetes mellitus, Chronic illness, Study validation, COSMIN

## Abstract

**Background:**

Worldwide, type 2 diabetes mellitus (T2DM) is one of the most prevalent chronic diseases and one of those producing greatest impact on patients’ day-to-day quality of life. Our study aim is to validate the “Living with Chronic Illness Scale” for a Spanish-speaking T2DM population.

**Methods:**

In this observational, international, cross-sectional study, 582 persons with T2DM were recruited in primary care and outpatient hospital consultations, in Spain and Colombia, during the period from May 2018 to June 2019. The properties analysed were feasibility/acceptability, internal consistency, reliability, precision and (structural) content-construct validity including confirmatory factor analysis. The COSMIN checklist was used to assess the methodological/psychometric quality of the instrument.

**Results:**

The scale had an adequate internal consistency and test retest reliability (Cronbach’s alpha = 0.90; intraclass correlation coefficient = 0.96, respectively). In addition, the instrument is precise (standard error of measurement = 3.34, with values < ½SD = 8.52) and correlates positively with social support (DUFSS) (r_s_ = 0.56), quality of life (WHOQOL-BREF) (r_s_ = 0.51–0.30) and ssatisfaction with life (SLS-6) (r_s_ = 0.50–0.38). The original 26-items version of the scale did not support totally the confirmatory factor analysis. The COSMIN checklist is favourable for all the properties analysed, although weaknesses are detected for structural validity.

**Conclusions:**

The LW-CI-T2DM is a valid, reliable and accurate instrument for use in clinical practice to determine how a person’s life is affected by the presence of diabetes. This instrument correlates well with the associated constructs of social support, quality of life and satisfaction. Additional research is needed to determine how well the questionnaire structure performs when robust factor analysis methods are applied.

**Supplementary Information:**

The online version contains supplementary material available at 10.1186/s12955-021-01715-x.

## Background

Human life expectancy has increased sharply during the last century, but in consequence chronic disease is more prevalent and patients are living longer with ill health [1]. Diabetes mellitus (DM) is one of the non-communicable diseases that most prejudices patients’ quality of life, and impacts directly on daily routines. In 2017, according to the International Diabetes Federation (IDF), 451 million people had DM, and this figure is expected to rise to 693 million by 2045. In the same year, five million deaths were attributed to DM, and worldwide 850 billion US dollars were spent on managing the disease [2]. The costs arising from DM are mainly due to the associated complications, chief among which are ischemic heart disease, stroke, diabetic foot and diabetic nephropathy [3, 4]. Socioeconomic factors such as gender, age, education, type of health insurance and the time of evolution since diagnosis are significantly associated with disease outcomes [5].

The impact of DM is not only physical but also psychosocial, as the disease requires comprehensive involvement by healthcare services, which must focus both on clinical symptoms and on how the patient copes with the disease [6]. In this field, organisations such as the European Association for the Study of Diabetes and the American Diabetes Association recommend that persons with type 2 diabetes mellitus (T2DM) should seek to achieve self-management on the basis of shared decision-making and on lifestyle modification [7].

Patients and healthcare providers do not always coincide in their perceptions of the disease, and therefore it is crucial to identify patients’ needs to ensure that appropriate clinical care is received. From this perspective, and in accordance with the steps outlined in Rodgers’ evolutionary concept analysis [8], we may consider the goal of living with one or more than one chronic illness, which has been defined as “*a complex, dynamic, cyclical and multidimensional process with the final desired target being to achieve positive living*” (page 7) [9]. Various instruments have been proposed to measure this or other concepts associated with managing chronicity. Some have evaluated specific constructs: for instance, the Minnesota Living with Heart Failure Questionnaire (MLHFQ) [10] and the Living with Chronic Obstructive Pulmonary Disease Questionnaire (LCOPD) [11] both address the patient’s quality of life; the Chronic Pain Acceptance Questionnaire (CPAQ) [12] focuses on acceptance of the process; Brief-COPE [13] considers how the patient copes with the situation. Other considerations are taken into account in the Diabetes Self-Management Questionnaire (DSMQ) [14] and the Psychosocial Adjustment to Illness Scale (PAIS) [15]. They focused on the evaluation of similar concepts related to the daily living like quality of life (MLHFQ, LCOPD), acceptance the chronic condition (CPAQ), coping the disease (Brief-COPE) or psychosocial adjustment to the disease (PAIS). However, none of these instruments evaluates the concept of living with a chronic illness such as T2DM. In this sense, one recently-developed scale addresses the concept of living with chronic illness, incorporating a broad spectrum of attributes, including acceptance, coping, self-management, integration and adaptation to the disease [9, 16]. This questionnaire, the Living with Chronic Illness (LW-CI) (from Spanish: *Escala de convivencia con un proceso crónico EC-PC*) scale includes 26 items spanning the above-mentioned dimensions. The LW-CI scale has been validated in a wide Spanish-speaking population with Parkinson’s disease and has adequate psychometric properties [17]. Its acceptability was later evaluated in a pilot study for other Spanish-speaking populations (with T2DM, heart failure -HF-, chronic obstructive pulmonary disease -COPD- or osteoarthritis), which showed that the instrument was viable and presented acceptable preliminary levels of validity [18]. The validation of this instrument for persons with T2DM will make it possible to identify the factors that determine whether a patient is living more or less positive with the disease, thus providing clinicians with valuable information enabling them to apply focused interventions.

## Methods

### Design

An observational, international and cross-sectional study (one point-in-time evaluation, with retest) was carried out. This study is part of a macro research project with the general aim to achieve a unique Spanish-speaking scale to evaluate the process of living with one or more than one long term condition (LTC), as T2DM, osteoarthritis, COPD, HF, high blood pressure, and Parkinson’s disease [17, 18]. In particular, the contribution of this manuscript in this field is to present the psychometric properties of the LW-CI-T2DM scale in a Spanish-speaking population.

### Sample, sampling and sample size

A consecutive cases sampling [19, 20] was applied to participant identification.

The sample was composed by people living with T2DM from different primary and secondary healthcare centres and community groups from Spain and Colombia. Inclusion criteria were (a) patients with T2DM diagnosis made by a endocrinology or General Practitioner (GP), in any stage of the disease; (b) Colombian or Spanish nationality; (c) able to read and understand properly the questionnaires; and (d) non-hospitalized patient at the moment of the study. Exclusion criteria were (a) patients with cognitive deterioration, acute disorder and/or pharmacological effects that potentially could distort the objective of the study; (b) refusal to participate in the study and (c) not meeting established inclusion criteria.

Sample size was calculated according to the model proposed by MacCallum-Browne-Sugawara [21]. To test a five-factor model, assuming the null hypothesis of a mean square error of approximation (RMSEA) from 0.05 to 0.08, a statistical power of 0.80, an alpha value of 0.05, with 205 df, a minimum sample size of 225 was required. This sample was over-estimated by 20% to cover possible losses. These calculations were carried out using STATISTICA 12 (Dell Software, Tulsa, OK).

### Instruments

A sociodemographic questionnaire was used to collect personal data of the patient living with T2DM related to gender, age, marital status, educational level and employment situation. Besides, T2DM related questionnaire was used in order to know age of diagnosis, disease duration, and type and duration of treatment for T2DM. As in other validation studies carried out in Spain and South America [17] in addition to sociodemographic and disease related data, the following Spanish versions of self-reported scales were also collected:

*LW-CI-T2DM* [18] scale focused to measure living with LTC. It is 26 items distributed in the following 5 dimensions: acceptance (4 items), coping (7 items), self-management (4 items), integration (5 items) and adjustment (6 items). It is a five-point Likert-scale ranging from 0 (nothing/never) to 4 (much/always) (except for the "acceptance” dimension, in which the score is reversed). The final score ranges from 0 and 104 (higher scores better living with the LTC).

- *The Duke-UNC Functional Social Support Questionnaire (DUFSS)* [22, 23] was used to evaluate social support of the patients’ from their perspective. It is an 8-items that evaluates different dimensions of social support as confidant, affective and instrumental support. The score for each item varies from 1 (much less than I would like) to 5 (as much as I would like).
- *The World Health Organization Quality of Life Instrument-Brief (WHOQOL-BREF)* [24] was used to measure the quality of life of people living with T2DM. The WHOQOL-BREF is comprised by 24-items that evaluates physical health, psychological health, social relationships, and environment. Item response options range from 1 (very dissatisfied) to 5 (very satisfied/very good quality of life).
- *The modified version of the Satisfaction with Life Scale* [25] is a to evaluate satisfaction overall with life (item 1) and in regard to other five areas: physical, psychological wellbeing, social relations, leisure, and financial situation. Each item scores from 0 (unsatisfied) to 10 (totally satisfied).
- *The Patient Based Global Impression of Severity Scale (PGIS)* [26] was used to evaluate the patient global impression of severity of the T2DM. It is a six-point Likert-scale ranging from 0 (not ill at all) to 5 (extremely ill) according to the patient.

For this validation study, the Spanish version of the scales was used.

### Data collection

Data collection was carried out between May 2018 and June 2019. The potential participants (people living with T2DM) filled in the scales during the consult with the endocrinology, GP, nurse specialist or primary care nurse. To ensure homogeneity and reproducibility of the procedure of data collection a standardized protocol was established with the following steps: explaining the research study; asking about doubts; reading out load instructions of the scales and its answer options; writing a check marc in the answer chosen by the patient; reading out load instructions of self-reported scales and giving participants time to complete it. The median time to complete all the measures was approximately 30–40 min.

Data collection related to test–retest was also protocolized to minimize potential random errors.

Patients were asked to answer a second time to the LW-CI-T2DM at home. The LW-CI-T2DM was in an envelope with seal and the research postal direction in order to complete the questionnaire and send it in an easy and free way to the researchers. A minimum sample of 50 subjects and a time span of 7 to 10 days for the retest was planned.

### Data analysis

Descriptive statistics (central tendency measures, proportions) were used to determine the sociodemographic and T2DM characteristics. Main data were ordinal or did not fit normal distribution. Therefore, non-parametric statistics were used.

For the following psychometric properties were tested in this LW-CI-T2DM validation study:

Feasibility and acceptability. Quality of data was considered satisfactory if 95% of the data were computable. The limit for missing data was < 5% [27]. Floor and ceiling effect were deemed acceptable if they were < 15% [28] and the skewness was expected between −1 to + 1 [29].

Internal consistency was tested by Cronbach’s alpha coefficient (criterion value > 0.70) [30], item-total correlation (corrected for overlap; criterion value, r_s_ ≥ 0.30) [31], inter-item correlation (criterion value, r ≥ 0.20 and ≤ 0.75) [32], and item homogeneity (criterion value > 0.30) [33].

Reproducibility (test–retest) was determined using weighted kappa (with quadratic weights) for items (standard: > 0.41 moderate) [34] and intraclass correlation coefficient (one way, random effect; ICC) for domains and total score. Values ≥ 0.60 were considered acceptable [35].

Precision was estimated by means of the standard error of measurement (SEM = SD_pooled_ * √ [1–r_xx_]), where $SD_{pooled } = \sqrt {\left( {SD_{1}^{2} + SD_{2}^{2} } \right)/2}$ and $r_{xx}$ was the ICC of the test–retest. A SEM value < ½SD_pooled_ was used as criterion of acceptable precision [36, 37].

Construct validity. Confirmatory factor analysis (CFA) was applied to corroborate the original 5-factor matrix of the instrument, using the following parameters of indices of good fit: (CMIN/df) less than 5 (preferably less than 3), RMSEA less than 0.08 with the respective 90% CI, comparative fit index (CFI) of more than 0.90, and goodness-of-fit index (GFI) of more than 0.90 [38]. CFA estimations were all standardized, which allowed that factor loading and covariance matrix to vary. For convergent validity, and according to a previous study of the scale in patients with Parkinson’s disease [14], a moderate (r_s_ ≥ 0.35–0.50) or strong relationship (r_s_ > 0.50) [39] was hypothesized between LW-CI-T2DM and DUFSS, SLS-6, and WHOQOL-BREF, and a weak/moderate association with other variables of the study, as age, T2DM duration or treatment. Spearman rank correlation coefficients were obtained to this purpose.

Internal validity, defined as the inter-correlations between the LW-CI-T2DM dimensions (standard, r_s_ = 0.30–0.70) [33] and known-groups validity for gender, treatment and PGIS scores were determined. Mann–Whitney and Kruskal–Wallis tests were used for groups comparison.

### COSMIN assessment

The COSMIN Checklist (COnsensus-based Standars for the selection of health Measurement INstruments) and its extension for content validity, were used for assessing the final measurement properties of the instrument and the methodology [40, 41].

## Results

**Fig. 1:**
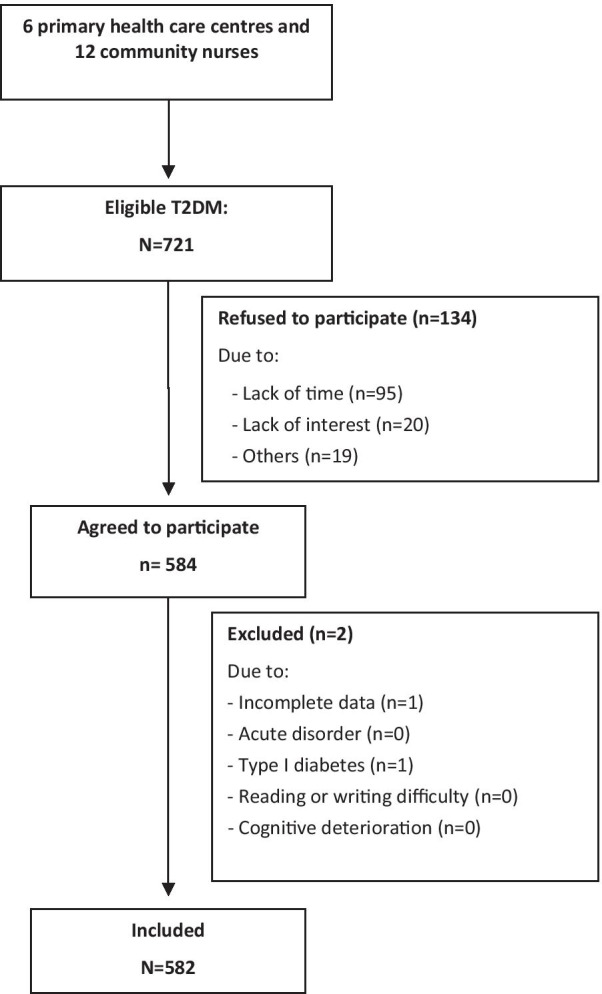
Flowchart of population sample

582 people living with T2DM from Spain and Colombia were included in this first validation study, where the 52.6% of the sample were females with an average age of 64.15 (SD = 2.18) (Fig. 1). The 57.5% were married, the 60.3% present primary studies showing a basic educational level and the 30% were actively working full time at the moment of the study. The mean age of T2DM duration was 10.25 (SD = 9.51) and the mean age with treatment 7.86 (SD = 7.80) (Table 1).

**Table 1:** Characteristics of sample

Sociodemographic characteristics	Total sample N = 582	Subsample Spain N = 248	Subsample Colombia N = 334	*P* value
Female (%)	306 (52.6)	95 (38.3)	211 (63.2)	< 0.001^a^
**Marital Status** (%)				
Single	87 (15.0)	34 (13.9)	53 (15.9)	< 0.001^a^
Married	333 (57.5)	165 (67.3)	168 (50.3)	
Widower	93 (16.1)	31 (12.7)	62 (18.6)	
Other	66 (11.4)	15 (6.1)	51 (15.3)	
**Occupation** (%)				< 0.001^a^
Active	174 (29.9)	79 (32.0)	95 (28.4)	
Homemaker	159 (27.4)	29 (11.7)	130 (38.9)	
Retired	121 (20.8)	116 (47.0)	5 (1.5)	
Other				
	127 (21.9)	23 (9.3)	104 (31.1)	
**Education** (%)				0.577^a^
None	11 (1.9)	3 (1.2)	8 (2.4)	
Primary	350 (60.3)	144 (58.5)	206 (61.7)	
High school	134 (23.1)	61 (24.8)	73 (21.9)	
University	85 (14.7)	38 (15.4)	47 (14.1)	
**Insulin therapy** (%)	268 (51.5)	69 (37.1)	199 (59.6)	< 0.001^a^
Age (mean ± SD)	64.15 (12.18)	65.27 (11.32)	63.32 (12,74)	0.056^b^
Time since diagnosis*	10.25 (9.51)	11.49 (10.54)	9.34 (8.56)	0.007 ^b^
Time with insulin therapy*	4.04 (7.81)	9.45 (12.16)	2.92 (6.01)	< 0.001^b^

*SD* standar desviation

*P* value between subsamples

^*^ Years

^a^ Chi test

^b^ Mann–Whitney-Wilcoxon test

Results related to data quality showed the scale had 6 missing data. There were 2 missing data in Domain 2. Coping, one missing data in Domain 3. Self-management, and 3 missing data in Domain 5. Adjustment. Regarding to acceptability, floor and ceiling effects for the total score ranged between 0.2 and 1.7% respectively. Domains and total score of the LW-CI-T2DM did not show skewness.

Results related to internal consistency of the LW-CI-T2DM showed that Cronbach’s alpha was 0.90 for the total scale and for the domains ranged between 0.71 (Domain 3. Self-management) and 0.82 (Domains 1. Acceptance and 5. Adjustment). Item homogeneity ranged between 0.36 (Domain 4. Integration) and 0.53 (Domain 1. Acceptance). As it is showed in Table 2, all corrected item-total correlations were higher than established standard value.

**Table 2:** Feasibility/Acceptability, reliability and precision of the LW-CI-T2DM scale

	LW-CI-T2DM scale					
	Domain 1. Acceptance	Domain 2. Coping	Domain 3. Self-management	Domain. 4 Integration	Domain 5. Adjustment	Total score
Mean (SD)	11.53 (3.74)	18.80 (5.56)	11.00 (3.37)	14.82 (3.66)	15.35 (5.45)	**71.53 (16.45)**
Data Quality (% fully computable data)	100	98	99	100	97	94
Floor effect (%)	1.0	0.5	0.7	0.2	0.5	0.2
Ceiling effect (%)	19.8	6.2	10.8	13.2	9.7	1.7
Skewness	−0.78	−0.47	−0.54	−0.60	−0–24	−0.34
Cronbach’s alpha	0.82	0.77	0.71	0.72	0.82	0.90
Item-total correlation	0.36–0.67	0.21–0.50	0.32–0.53	0.16–0.58	0.16–0.60	–
Item homogeneity	0.53	0.33	0.40	0.36	0.42	–
Reproducibility (ICC)	0.93	0.93	0.90	0.91	0.96	0.96
Precision (SEM) (1/2 SD)	0.92 (1.74)	1.52 (2.88)	1.05 (1.65)	1.01 (1.68)	1.08 (2.71)	3.34 (8.52)

*LW-CI-T2DM:* Living with type 2 diabetes mellitus scale

*SD* standard deviation, *ICC *intraclass correlation coefficient, *SEM* standard error of measurement = $SD_{pooled} *\surd \left( {1 - ICC} \right)$.

Regarding the reproducibility of the LW-CI-T2DM (test–retest), this analysis was carried out in 135 patients living with T2DM. The ICC for the total scale was 0.96 and for all domains over 0.90 (Table 3). For individual items, weighted kappa ranged between 0.66 (item 24) and 0.88 (items 22).

**Table 3:** Convergent validity and internal validity of LW-CI-T2DM scale

	LW-CI-T2DM scale					
	Domain 1. Acceptance	Domain 2. Coping	Domain 3. Self-management	Domain 4. Integration	Domain 5. Adjustment	Total score
**Convergent validity**						
Age	0.14**	−0.04	−0.02	0.05	−0.05	0.02
Age onset T2DM	0.14**	−0.1*	−0.02	0.06	−0.02	0.04
T2DM duration	0.09*	−0.08	−0.01	−0.02	−0.04	−0.10
WHOQOL—Physical health	0.22**	0.22**	0.21**	0.31**	0.24**	0.30**
WHOQOL—Psychological	0.23**	0.44**	0.40**	0.48**	0.45**	0.51**
WHOQOL—Social relationships	0.19**	0.31**	0.27**	0.40**	0.32**	0.38**
WHOQOL—Environment	0.22**	0.32**	0.34**	0.49**	0.34**	0.43**
DUFSS	0.18**	0.48**	0.46**	0.49**	0.47**	0.56**
Satisfaction With Life	0.28**	0.34**	0.36**	0.47**	0.40**	0.50**
Satisfaction—physical health	0.24**	0.31**	0.30**	0.42**	0.38**	0.44**
Satisfaction—well−being	0.26**	0.25**	0.24**	0.32**	0.35**	0.38**
Satisfaction—social relations	0.29**	0.33**	0.31**	0.39**	0.35**	0.45**
Satisfaction—leisure	0.26**	0.31**	0.33**	0.39**	0.31**	0.48**
Satisfaction—financial situation	0.19**	0.35**	0.30**	0.41**	0.29**	0.41**
**Internal validity**						
Coping	0.09*	–	–	–	–	–
Self-management	0.17**	0.61**	–	–	–	–
Integration	0.24**	0.62**	0.65**	–	–	–
Adjustment	0.13**	0.62**	0.56**	0.59**	–	–

*LW-CI-T2DM scale* Living with type 2 diabetes mellitus scale

**p < 0.01; *p < 0.05

SEM was 3.34 (< ½SD = 8.52) for the total score of the scale and for the domains range from 0.92 to 1.52 (see Table 3).

Related to convergent validity, the LW-CI-T2DM presented strong relationship with DUFSS (*r*_*s*_ = 0.56), with SLS-6 (*r*_*s*_ = 0.50) and with Domain 2 of WHOQOL-BREF related to psychological health of the person (*r*_*s*_ = 0.51). Besides, the LW-CI-T2DM presented moderate correlations with all items of the SLS-6 and Domains 3 and 4 of the WHOQOL-BREF related to social relationships and environment, respectively. Weak correlation was found with T2DM duration and physical health of the patient (see Table 3). According to internal validity, domains inter-correlated from 0.09 (Acceptance with Coping) to 0.65 (Self-management and Integration). See Table 3 for further detail. In relation to known-group validity, results showed that total scores were significantly different for gender (higher in women) and for PGIS (see Table 4).

**Table 4:** Known-group validity

Categories	LW-CI-T2DM total	*P* value
**Sex**		
Men	68.65 (16.21)	< 0.001
Women	74.12 (16.28)	
**Treatment for T2DM (insulin)**		
Yes	72.45 (16.49)	0.09
No	69.75 (16.70)	
**PGIS-based severity levels**		
None	75.70 (17.00)	< 0.001
Mild	71.31 (15.65)	
Moderate	71.52 (15.61)	
Severe	64.27 (17.10)	

Mean (standard deviation)

*LW-CI-T2DM* Living with type 2 diabetes mellitus scale

The CFA did not support the original 5-dimension model with 26 items. However, after analyzing residual errors in the standardized matrix of residual covariance, a final structure with 5 factor and 23 items showed better fit. Good indices were obtained: CMIN/*df* = 3.11; goodness of fit index = 0.91; comparative fit index = 0.91 and root mean square error of approximation = 0.06 (90% confidence interval, 0.06–0.07) (a complete description of the fit is available as Additional file 1).

### COSMIN results

Five psychometric properties are addressed in the LW-CI-T2DM scale (Boxes A-E of the COSMIN checklist). The instrument obtains acceptable results for internal consistency, measurement error, reliability and structural (construct) validity. The content validity lacked information from patients and healthcare professionals. This shortcoming is reflected in the COSMIN extension on the evaluation of the development quality of the Patient Reported Outcome (PRO) (a full description is available as Additional file 2).

## Discussion

The aim of this study is to validate the Spanish version of the LW-CI scale for persons with T2DM, obtaining an instrument (LW-CI-T2DM) to measure how this population lives with the disease, with study outcomes that are useful both for researchers and in clinical practice.

Members of the research team have been working for several years to best define the concept of living with a chronic illness. For this purpose, an in-depth conceptual analysis was first performed [9], reviewing the literature on this question and making use of Rodgers’ method of evolutionary concept analysis [8]. Before the psychometric evaluation, the questionnaire was piloted in various populations of persons with at least one chronic disease (including T2DM), to determine its viability and acceptability [18]. Many experts in the field consider this practice essential to ensure that the questionnaire items really address the construct that is to be measured [42].

In the present study, the non-response rate was less than 5% for all dimensions; there was no floor effect and in relation to the ceiling effect, only the 15% limit was exceeded, and that very slightly, for the “acceptance” dimension. These data suggest that, a priori, the scale provides reasonable acceptability [28].

LW-CI-T2DM has excellent internal consistency (Cronbach’s alpha = 0.90) both overall and for each dimension, always remaining within the recommended limits, which suggests there is no redundancy in the content of the questions [43]. Similarly, the questionnaire presents high reliability in the sub-sample selected for the retest, comfortably surpassing the minimum levels recommended (ICC > 0.70) [43] despite its significant extension, with 26 items. These findings suggest that LW-CI-T2DM is a parsimonious instrument, measuring the intended aspects of the question with the fewest items possible, a quality that is highly desirable [44].

The CFA did not support the original structure of the designed scale based on solid conceptual framework [9] and other validation studies carried out in patient with other chronic conditions such as Parkinson’s disease [17]. A 23-item version adjusted better than the 26-item original version. However, based on previous conceptual and empirical findings [9, 17, 18] as well as a research team consensus, further research to elucidate the final configuration of the scale, using a robust validation methodology such as Rasch analysis [45] is suggested. Nevertheless, the results emerged in this validation study will be carefully considered in further studies carried out in patient living with different chronic conditions, such as T2DM among others.

Finally, the instrument is precise and correlates positively, at least to a moderate degree, with the existence of social support and with each of the subdimensions of the scales measuring satisfaction and quality of life. The correlation data are similar to those reported for the population with Parkinson’s disease [17], showing that the questionnaire measures these cross-sectional constructs in a similar way in each of these chronic conditions. However, we must consider this aspect with caution, because the measurement invariance between groups has not yet been established.

Application of the LW-CI-T2DM scale reveals significant differences according to the severity of the condition; thus, patients who are assigned higher scores (reflecting better coexistence with the disease) tend to be those who are less severely affected. Moreover, these scores are generally higher than those obtained by persons with Parkinson’s disease, suggesting that living with T2DM is more tolerable. Differences by gender were also obtained, with higher scores for women. This finding differs from that produced by the pilot study, although this preliminary work included a population with other chronic diseases (COPD, HF or osteoarthritis) [18]. Other studies have shown that women with T2DM are at greater risk than men of psychosocial maladjustment, a poorer cardiovascular profile and/or non-compliance with treatment goals [46, 47]. These outcomes are not consistent with our findings and further research is needed to clarify the question.

From a conceptual standpoint, the LW-CI-T2DM scale has similarities with constructs addressed by other theoretical models. Thus, dimensions such as self-management or coping bear an important relationship with Bandura’s concept of self-efficacy [48], which is widely used by other instruments in psychosocial approaches to chronic diseases [49, 50]. Other dimensions, such as integration or adaptation, are closely linked to the notion of perceived control, introduced by Ajzen in his Theory of Planned Behaviour (TPB) [51]. The instrument also correlates very reasonably with social support, a concept also introduced in the TPB as the subjective norm (perceptions of the impact of third parties—such as family, friends or healthcare professionals—on whether or not the conduct in question takes place). The use of a conceptual model to underpin the LW-CI-T2DM instrument enables the analyst to explain inductively how events happen and to suggest practical solutions to the problems encountered.

At the clinical level, the value of the instrument lies in its explanatory capacity, reflecting how a person with T2DM lives with the disease and thus allowing professionals to focus on the most troublesome aspects. This role is especially significant because healthcare professionals commonly express frustration at not achieving the expected results from treatment and recommendations. On the other hand, many patients believe their healthcare is not sufficiently individualised [52]. Prior analyses of patients with Parkinson’s disease have shown that social support, followed by satisfaction with life and by socioeconomic status, are the only factors relevant to the patient’s coexistence with the disease [53]. If these factors were equally influential with respect to T2DM, we would be facing a scenario in which social factors exerted significant influence on health conditions and should be taken into account when socio-health policies are designed and applied.

Although the present study has been performed with all possible rigour, it is subject to certain limitations. According to the International Society for Pharmacoeconomics and Outcomes Research (ISPOR), for an instrument to present content validity, it must obtain information derived from what is already known on the subject, from the reference population and from healthcare providers [54]. While LW-CI-T2DM is based on an important conceptual analysis of published research, the findings have not been triangulated with information obtained by qualitative techniques (via focus groups, cognitive interviews, the Delphi method, etc.), as described in the relevant section of the COSMIN checklist (Additional file 2). Furthermore, due to the innovative nature of the concept, LW-CI-T2DM lacks a gold standard with which to verify its criteria validity. However, other instruments have been proposed to evaluate psychosocial aspects of diabetes, such as the Problem Areas in Diabetes [55] and the Diabetes Empowerment Scale [56]. Although the approach they take is different from our own, it might be useful to analyse their possible correlations with LW-CI-T2DM scale. Finally, due to the intrinsic nature of the present research, the question of sensitivity to change has not been evaluated and as it is showed in COSMIN checklist (Additional file 2) structural validity have some flaws due to confirmatory factor analysis indices were not totally adequate.

## Conclusions

LW-CI-T2DM is a valid, reliable and precise instrument for assessing the question of living with T2DM. Additional research is needed to identify the factors that specifically impact on the concept of “living with” this disease. In addition, more extensive analyses should be made of the construct under study, by robust methods such as evaluating its factor structure by means of structural equations.

## Supplementary Information

**Additional file 1**. Confirmatory factor analysis process.

**Additional file 2**. COSMIN assessment.

## Data Availability

The datasets used and/or analysed during the current study are available from the corresponding author on reasonable request.

## Abbreviations

T2DM: Type 2 Diabetes Mellitus
COSMIN: Consensus-based Standars for the selection of health Measurement Instruments
DUFSS: Duke-UNC Functional Social Support Scale
WHOQOL-BREF: World Health Organization Quality of Life Instrument-Brief Scale
SLS-6: Satisfaction with Life Scale
PGIS: Patient Based Global Impression of Severity Scale
LW-CI-T2DM: Living With Type 2 Diabetes Mellitus Scale
IDF: International Diabetes Federation
MLHFQ: Minnesota Living with Heart Failure Questionnaire
LCOPD: Living with Chronic Obstructive Pulmonary Disease questionnaire
CPAQ: Chronic Pain Acceptance Questionnaire
DSMQ: Diabetes Self-Management Questionnaire
PAIS: Psychosocial Adjustment to Illness Scale
LTC: Long term condition
GP: General Practitioner
ICC: Intraclass correlation coefficient
SEM: Standard error of measurement
SD: Standard desviation
PRO: Patient Reported Outcome
HF: Heart failure
COPD: Chronic obstructive pulmonary disease
TPB: Theory of Planned Behavior
PD: Parkinson’s Disease
ISPOR: International Society for Pharmacoeconomics and Outcomes Research
