# Supplementary material for: Validation of living with chronic illness scale in a type 2 diabetes mellitus population
Source: Health Qual Life Outcomes. 2021 Mar 17;19:93. doi: 10.1186/s12955-021-01715-x (PMC7972215; doi:10.1186/s12955-021-01715-x)
Supplement: Supplementary file 1 — Additional file 1. Confirmatory factor analysis process. [file 12955_2021_1715_MOESM1_ESM.docx]

**Validation of Living with Chronic Illness Scale in a Type 2 Diabetes Mellitus population**

Additional material

ADDITIONAL FILE 1. CONFIRMATORY FACTOR ANALISYS PROCESS 3

ADDITIONAL FILE 2. COSMIN ASSESSMENT 7

Step 1. Evaluated measurement properties in the article 7

Step 2. Determining if the statistical method used in the article are based on CTT or IRT 7

Step 3. Determining if a study meets the standards for good methodological quality 8

Box A. Internal consistency 8

Box C. Measurement error: absolute measures 10

Box E. Structural validity 11

Step 4: Determining the Generalisability of the results 12

COSMIN box 1. Standards for evaluating the quality of PROM development 13

Box 1. PROM development 13

COSMIN box 2. Standards for evaluating the quality of content validity studies of PROMs 16

Box 2. Content validity 16

# ADDITIONAL FILE 1. CONFIRMATORY FACTOR ANALISYS PROCESS

1º) Original version with 26 items


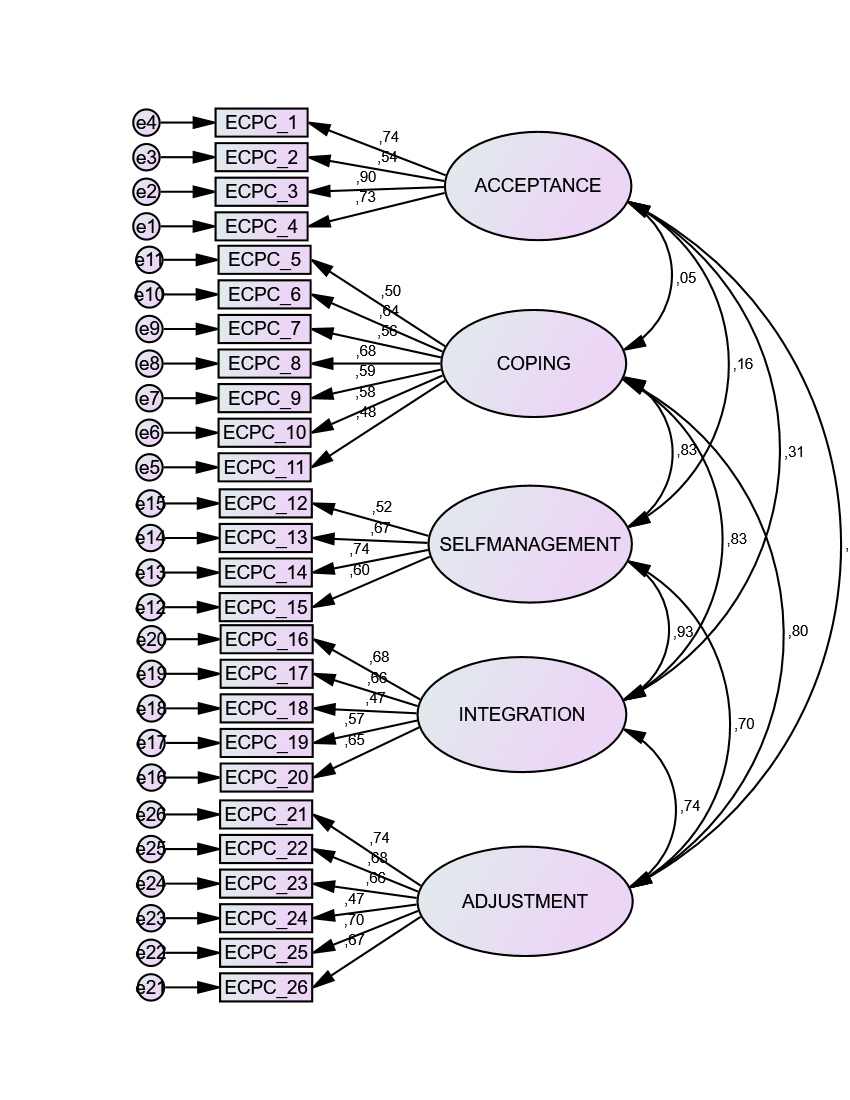


CMIN/*df=* 4.81; goodness of fit index= 0.82; comparative fit index= 0.82 and root mean square error of approximation = 0.08 (90% confidence interval, 0.08-0.09)

2º) Version with 25 items (item 24 removed)


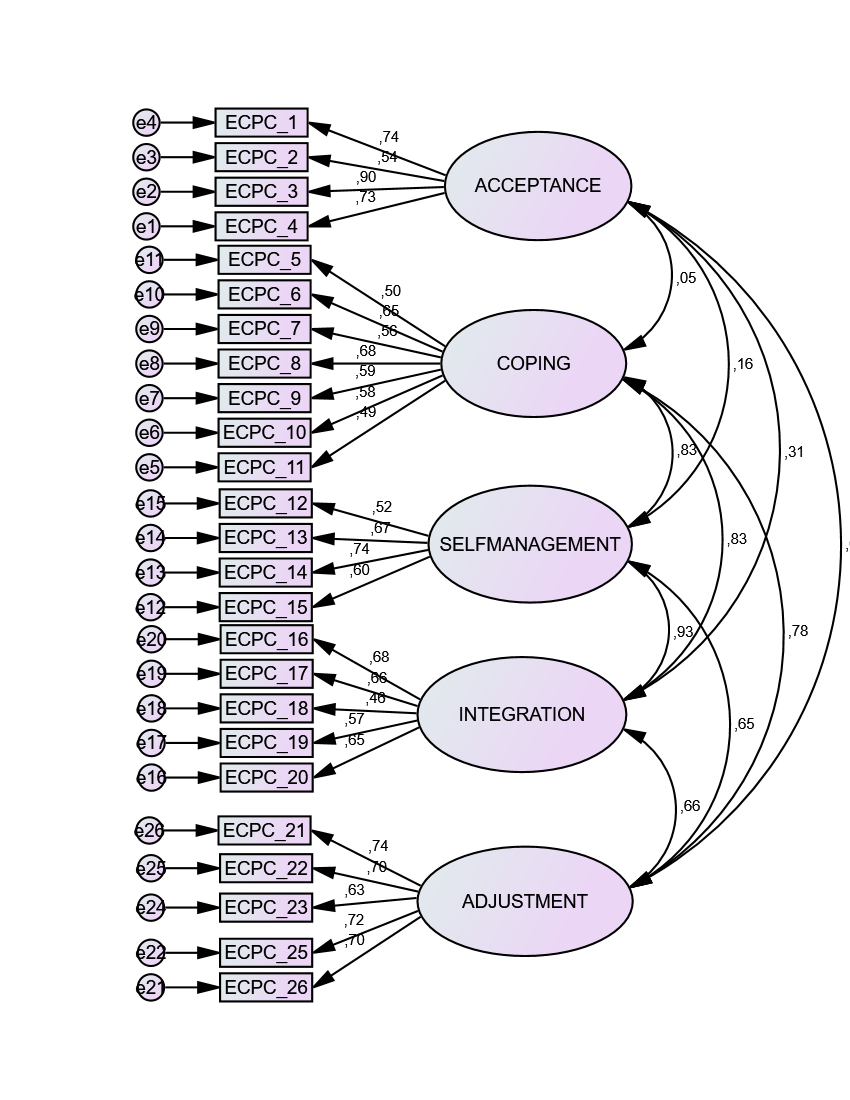


CMIN/*df=* 4.08; goodness of fit index= 0.86; comparative fit index= 0.85 and root mean square error of approximation = 0.07 (90% confidence interval, 0.07-0.08)

3º) Version with 25 items (item 24 removed) with constrictions


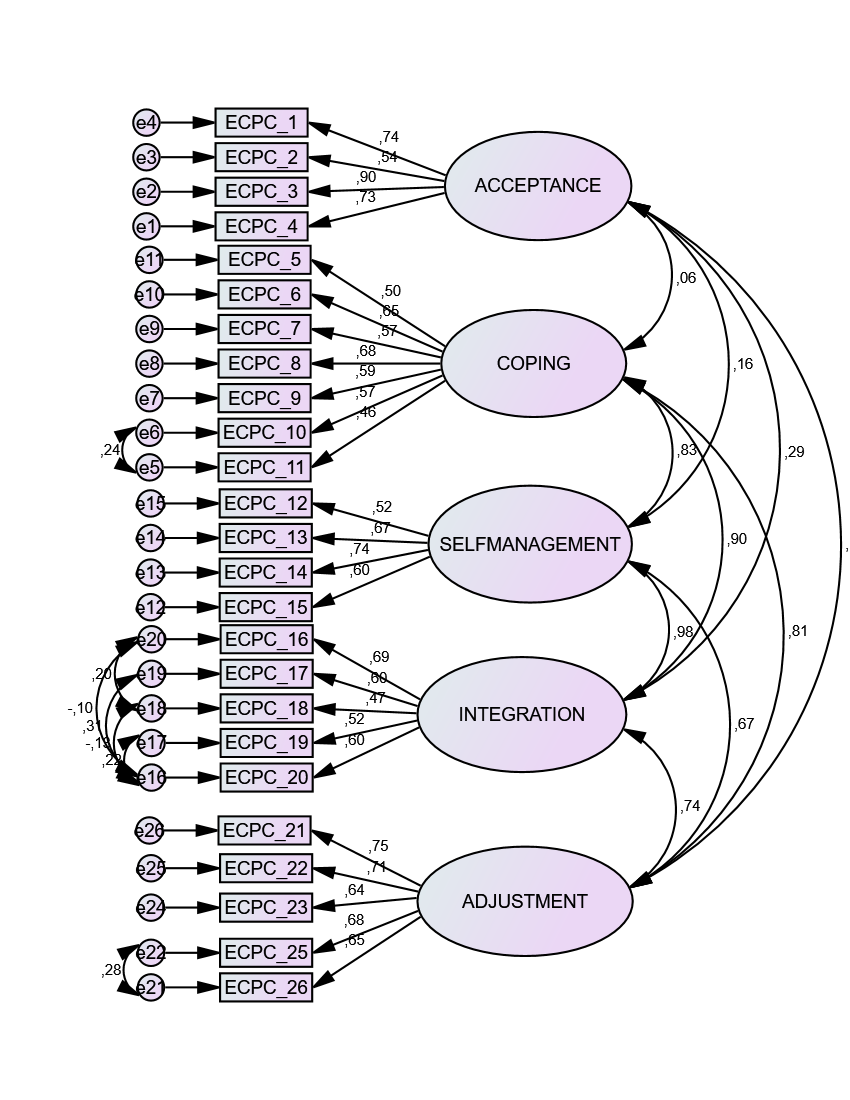


CMIN/*df=* 3.43; goodness of fit index= 0.89; comparative fit index= 0.89 and root mean square error of approximation = 0.06 (90% confidence interval, 0.06-0.07)

4º) Final version with 23 items (items 11 and 18 removed for correlation < 0,5)


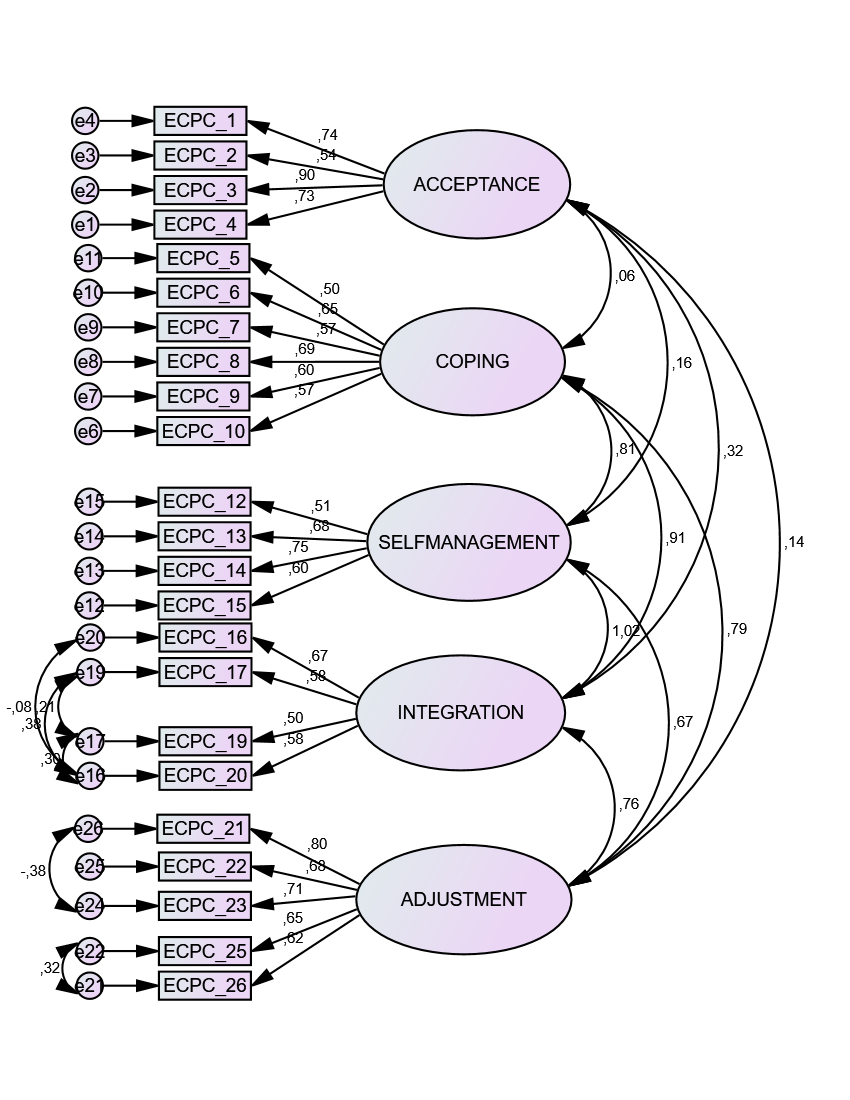


CMIN/*df=* 3.11; goodness of fit index= 0.91; comparative fit index= 0.91 and root mean square error of approximation = 0.06 (90% confidence interval, 0.06-0.07)
