# Supplementary material for: Validation of living with chronic illness scale in a type 2 diabetes mellitus population
Source: Health Qual Life Outcomes. 2021 Mar 17;19:93. doi: 10.1186/s12955-021-01715-x (PMC7972215; doi:10.1186/s12955-021-01715-x)
Supplement: Supplementary file 2 — Additional file 2. COSMIN assessment. [file 12955_2021_1715_MOESM2_ESM.docx]

# ADDITIONAL FILE 2. COSMIN ASSESSMENT

##

## Step 1. Evaluated measurement properties in the article

| X | Internal consistency | Box A |
| --- | --- | --- |
| X | Reliability | Box B |
| X | Measurement error | Box C |
| X | Content validity | Box D |
| X | Structural validity | Box E |
|  | Hypotheses testing | Box F |
|  | Cross-cultural validity | Box G |
|  | Criterion validity | Box H |
|  | Responsiveness | Box I |
|  | Interpretability | Box J |

## Step 2. Determining if the statistical method used in the article are based on CTT or IRT

|  | **Box General requirements for studies that applied Item Response Theory (IRT) models** | | | | | | |
| --- | --- | --- | --- | --- | --- | --- | --- |
|  |  | **yes** |  | **no** |  | **?** |  |
| 1 | Was the IRT model used adequately described? e.g. One Parameter Logistic Model (OPLM), Partial Credit Model (PCM), Graded Response Model (GRM) |  |  | X |  |  |  |
|  |  |  |  |  |  |  |  |
|  |  |  |  |  |  |  |  |
| 2 | Was the computer software package used adequately described? e.g. RUMM2020, WINSTEPS, OPLM, MULTILOG, PARSCALE, BILOG, NLMIXED |  |  | X |  |  |  |
|  |  |  |  |  |  |  |  |
|  |  |  |  |  |  |  |  |
| 3 | Was the method of estimation used adequately described? e.g. conditional maximum likelihood (CML), marginal maximum likelihood (MML) |  |  | X |  |  |  |
|  |  |  |  |  |  |  |  |
|  |  |  |  |  |  |  |  |
| 4 | Were the assumptions for estimating parameters of the IRT model checked? e.g. unidimensionality, local independence, and item fit (e.g. differential item functioning (DIF)  *The model used in the LW-T2DM scale is based on Classical Test Theory |  |  |  |  | X |  |
|  |  |  |  |  |  |  |  |
|  |  |  |  |  |  |  |  |
|  |  |  |  |  |  |  |  |

## Step 3. Determining if a study meets the standards for good methodological quality

|  | Box A. Internal consistency |  |  |  |  | |  | | |  | | |  | | |  | |
| --- | --- | --- | --- | --- | --- | --- | --- | --- | --- | --- | --- | --- | --- | --- | --- | --- | --- |
|  |  | **yes** |  | **no** |  | | **?** | | |  | | |  | | |  | |
| 1 | Does the scale consist of effect indicators, i.e. is it based on a reflective model? | X |  |  |  | |  | | |  | | |  | | |  | |
| *Design requirements* | | **yes** |  | **no** |  | | **?** | | |  | | |  | | |  | |
| 2 | Was the percentage of missing items given? | X |  |  |  | |  | | |  | | |  | | |  | |
|  |  |  |  |  |  | |  | | |  | | |  | | |  | |
| 3 | Was there a description of how missing items were handled? | X |  |  |  | |  | | |  | | |  | | |  | |
|  |  |  |  |  |  | |  | | |  | | |  | | |  | |
| 4 | Was the sample size included in the internal consistency analysis adequate? | X |  |  |  | |  | | |  | | |  | | |  | |
|  |  |  |  |  |  | |  | | |  | | |  | | |  | |
| 5 | Was the unidimensionality of the scale checked? i.e. was factor analysis or IRT |  |  | X |  | |  | | |  | | |  | | |  | |
|  | model applied? |  |  |  |  | |  | | |  | | |  | | |  | |
|  |  |  |  |  |  | |  | | |  | | |  | | |  | |
| 6 | Was the sample size included in the unidimensionality analysis adequate? |  |  | X |  | |  | | |  | | |  | | |  | |
|  |  |  |  |  |  | |  | | |  | | |  | | |  | |
| 7 | Was an internal consistency statistic calculated for each (unidimensional) (sub)scale | X |  |  |  | |  | | |  | | |  | | |  | |
|  | separately? |  |  |  |  | |  | | |  | | |  | | |  | |
| 8 | Were there any important flaws in the design or methods of the study? |  |  | X |  | |  | | |  | | |  | | |  | |
| *Statistical methods* | | **yes** |  | **no** |  | | **NA** | | |  | | |  | | |  | |
| 9 | for Classical Test Theory (CTT): Was Cronbach’s alpha calculated? | X |  |  |  | |  | | |  | | |  | | |  | |
|  |  |  |  |  |  | |  | | |  | | |  | | |  | |
| 10 | for dichotomous scores: Was Cronbach’s alpha or KR-20 calculated? |  |  |  |  | | X | | |  | | |  | | |  | |
|  |  |  |  |  |  | |  | | |  | | |  | | |  | |
| 11 | for IRT: Was a goodness of fit statistic at a global level calculated? e.g. χ2, reliability |  |  |  |  | | X | | |  | | |  | | |  | |
|  | coefficient of estimated latent trait value (index of (subject or item) separation) |  |  |  |  | |  | | |  | | |  | | |  | |
|  |  |  |  |  |  | |  | | |  | | |  | | |  | |
|  | Box B. Reliability: relative measures **(including test-retest reliability, inter-rater reliability and intra-rater reliability)** | | | | | | | | | | | | | | | | |
| *Design requirements* | | | | | | **yes** | |  | **no** | |  | **?** | |  |  | |  |
| 1 | Was the percentage of missing items given? | | | | |  | |  | X | |  |  | |  |  | |  |
|  |  | | | | |  | |  |  | |  |  | |  |  | |  |
| 2 | Was there a description of how missing items were handled? | | | | |  | |  | X | |  |  | |  |  | |  |
|  |  | | | | |  | |  |  | |  |  | |  |  | |  |
| 3 | Was the sample size included in the analysis adequate? | | | | | X | |  |  | |  |  | |  |  | |  |
|  |  | | | | |  | |  |  | |  |  | |  |  | |  |
| 4 | Were at least two measurements available? | | | | | X | |  |  | |  |  | |  |  | |  |
|  |  | | | | |  | |  |  | |  |  | |  |  | |  |
| 5 | Were the administrations independent? | | | | | X | |  |  | |  |  | |  |  | |  |
|  |  | | | | |  | |  |  | |  |  | |  |  | |  |
| 6 | Was the time interval stated? | | | | | X | |  |  | |  |  | |  |  | |  |
|  |  | | | | |  | |  |  | |  |  | |  |  | |  |
| 7 | Were patients stable in the interim period on the construct to be measured? | | | | |  | |  |  | |  | X | |  |  | |  |
|  |  | | | | |  | |  |  | |  |  | |  |  | |  |
| 8 | Was the time interval appropriate? | | | | | X | |  |  | |  |  | |  |  | |  |
|  |  | | | | |  | |  |  | |  |  | |  |  | |  |
| 9 | Were the test conditions similar for both measurements? e.g. type of | | | | | X | |  |  | |  |  | |  |  | |  |
|  | administration, environment, instructions | | | | |  | |  |  | |  |  | |  |  | |  |
| 10 | Were there any important flaws in the design or methods of the study? | | | | |  | |  | X | |  |  | |  |  | |  |
| *Statistical methods* | | | | | | **yes** | |  | **no** | |  | **NA** | |  | **?** | |  |
| 11 | for continuous scores: Was an intraclass correlation coefficient (ICC) calculated? | | | | | X | |  |  | |  |  | |  |  | |  |
|  |  | | | | |  | |  |  | |  |  | |  |  | |  |
| 12 | for dichotomous/nominal/ordinal scores: Was kappa calculated? | | | | | X | |  |  | |  |  | |  |  | |  |
|  |  | | | | |  | |  |  | |  |  | |  |  | |  |
| 13 | for ordinal scores: Was a weighted kappa calculated? | | | | | X | |  |  | |  |  | |  |  | |  |
|  |  | | | | |  | |  |  | |  |  | |  |  | |  |
| 14 | for ordinal scores: Was the weighting scheme described? e.g. linear, quadratic | | | | |  | |  | X | |  |  | |  |  | |  |
|  |  | | | | |  | |  |  | |  |  | |  |  | |  |

|  | Box C. Measurement error: absolute measures |  |  |  |  |  |  |  |  |
| --- | --- | --- | --- | --- | --- | --- | --- | --- | --- |
|  |  |  |  |  |  |  |  |  |  |
| *Design requirements* | | **yes** |  | **no** |  | **?** |  |  |  |
|  |  |  |  |  |  |  |  |  |  |
| 1 | Was the percentage of missing items given? | X |  |  |  |  |  |  |  |
|  |  |  |  |  |  |  |  |  |  |
| 2 | Was there a description of how missing items were handled? | X |  |  |  |  |  |  |  |
|  |  |  |  |  |  |  |  |  |  |
| 3 | Was the sample size included in the analysis adequate? | X |  |  |  |  |  |  |  |
|  |  |  |  |  |  |  |  |  |  |
| 4 | Were at least two measurements available? | X |  |  |  |  |  |  |  |
|  |  |  |  |  |  |  |  |  |  |
| 5 | Were the administrations independent? | X |  |  |  |  |  |  |  |
|  |  |  |  |  |  |  |  |  |  |
| 6 | Was the time interval stated? | X |  |  |  |  |  |  |  |
|  |  |  |  |  |  |  |  |  |  |
| 7 | Were patients stable in the interim period on the construct to be measured? | X |  |  |  |  |  |  |  |
|  |  |  |  |  |  |  |  |  |  |
| 8 | Was the time interval appropriate? | X |  |  |  |  |  |  |  |
|  |  |  |  |  |  |  |  |  |  |
| 9 | Were the test conditions similar for both measurements? e.g. type of | X |  |  |  |  |  |  |  |
|  | administration, environment, instructions |  |  |  |  |  |  |  |  |
|  |  |  |  |  |  |  |  |  |  |
| 10 | Were there any important flaws in the design or methods of the study? |  |  | X |  |  |  |  |  |
|  |  |  |  |  |  |  |  |  |  |
| *Statistical methods* | | **yes** |  | **no** |  | **?** |  |  |  |
|  |  |  |  |  |  |  |  |  |  |
| 11 | for CTT: Was the Standard Error of Measurement (SEM), Smallest Detectable Change | X |  |  |  |  |  |  |  |
|  | (SDC) or Limits of Agreement (LoA) calculated? |  |  |  |  |  |  |  |  |
|  |  |  |  |  |  |  |  |  |  |

|  | Box D. Content validity **(including face validity)** |  |  |  |  |  |  |  |  |
| --- | --- | --- | --- | --- | --- | --- | --- | --- | --- |
| *General requirements* | | **yes** |  | **no** |  | **?** |  |  |  |
|  |  |  |  |  |  |  |  |  |  |
| 1 | Was there an assessment of whether all items refer to relevant aspects of the | X |  |  |  |  |  |  |  |
|  | construct to be measured? |  |  |  |  |  |  |  |  |
|  |  |  |  |  |  |  |  |  |  |
| 2 | Was there an assessment of whether all items are relevant for the study | X |  |  |  |  |  |  |  |
|  | population? (e.g. age, gender, disease characteristics, country, setting) |  |  |  |  |  |  |  |  |
|  |  |  |  |  |  |  |  |  |  |
| 3 | Was there an assessment of whether all items are relevant for the purpose of the |  |  |  |  | X |  |  |  |
|  | measurement instrument? (discriminative, evaluative, and/or predictive) |  |  |  |  |  |  |  |  |
|  |  |  |  |  |  |  |  |  |  |
| 4 | Was there an assessment of whether all items together comprehensively reflect the | X |  |  |  |  |  |  |  |
|  | construct to be measured? |  |  |  |  |  |  |  |  |
|  |  |  |  |  |  |  |  |  |  |
| 5 | Were there any important flaws in the design or methods of the study? | X |  |  |  |  |  |  |  |
|  |  |  |  |  |  |  |  |  |  |

|  | Box E. Structural validity |  |  |  |  |  |  |  |  |
| --- | --- | --- | --- | --- | --- | --- | --- | --- | --- |
|  |  | **yes** |  | **no** |  | **?** |  |  |  |
| 1 | Does the scale consist of effect indicators, i.e. is it based on a reflective model? | X |  |  |  |  |  |  |  |
|  |  |  |  |  |  |  |  |  |  |
| *Design requirements* | | **yes** |  | **no** |  | **?** |  |  |  |
|  |  |  |  |  |  |  |  |  |  |
| 2 | Was the percentage of missing items given? | X |  |  |  |  |  |  |  |
|  |  |  |  |  |  |  |  |  |  |
| 3 | Was there a description of how missing items were handled? | X |  |  |  |  |  |  |  |
|  |  |  |  |  |  |  |  |  |  |
| 4 | Was the sample size included in the analysis adequate? | X |  |  |  |  |  |  |  |
|  |  |  |  |  |  |  |  |  |  |
| 5 | Were there any important flaws in the design or methods of the study? | X |  |  |  |  |  |  |  |
|  |  |  |  |  |  |  |  |  |  |
| *Statistical methods* | | **yes** |  | **no** |  | **NA** |  |  |  |
|  |  |  |  |  |  |  |  |  |  |
| 6 | for CTT: Was exploratory or confirmatory factor analysis performed? | X |  |  |  |  |  |  |  |
|  |  |  |  |  |  |  |  |  |  |
| 7 | for IRT: Were IRT tests for determining the (uni-) dimensionality of the items |  |  |  |  | X |  |  |  |
|  | performed? |  |  |  |  |  |  |  |  |
|  |  |  |  |  |  |  |  |  |  |

## Step 4: Determining the Generalisability of the results

|  | **Box Generalisability** |  |  |  |  |  |  |  |  |
| --- | --- | --- | --- | --- | --- | --- | --- | --- | --- |
|  |  | **yes** |  | **no** |  | **NA** |  |  |  |
| Was the sample in which the HR-PRO instrument was evaluated adequately described? | |  |  |  |  |  |  |  |  |
| In terms of: | |  |  |  |  |  |  |  |  |
|  |  |  |  |  |  |  |  |  |  |
| 1 | median or mean age (with standard deviation or range)? | X |  |  |  |  |  |  |  |
|  |  |  |  |  |  |  |  |  |  |
| 2 | distribution of sex? | X |  |  |  |  |  |  |  |
|  |  |  |  |  |  |  |  |  |  |
| 3 | important disease characteristics (e.g. severity, status, duration) and description |  |  |  |  | X |  |  |  |
|  | of treatment? |  |  |  |  |  |  |  |  |
|  |  |  |  |  |  |  |  |  |  |
| 4 | setting(s) in which the study was conducted? e.g. general population, primary care | X |  |  |  |  |  |  |  |
|  | or hospital/rehabilitation care |  |  |  |  |  |  |  |  |
|  |  |  |  |  |  |  |  |  |  |
| 5 | countries in which the study was conducted? | X |  |  |  |  |  |  |  |
|  |  |  |  |  |  |  |  |  |  |
| 6 | language in which the HR-PRO instrument was evaluated? | X |  |  |  |  |  |  |  |
|  |  |  |  |  |  |  |  |  |  |
| 7 | Was the method used to select patients adequately described? e.g. convenience, | X |  |  |  |  |  |  |  |
|  | consecutive, or random |  |  |  |  |  |  |  |  |
|  |  | **yes** |  | **no** |  | **?** |  |  |  |
|  |  |  |  |  |  |  |  |  |  |
| 8 | Was the percentage of missing responses (response rate) acceptable? | X |  |  |  |  |  |  |  |
|  |  |  |  |  |  |  |  |  |  |

## COSMIN box 1. Standards for evaluating the quality of PROM development

Part 1a (PROM design) and part 1b (Cognitive interview study or other pilot test) both need to be completed for each PROM because all standards of part 1a and part 1b will be

included in the final rating of the quality of the PROM development. However, if a cognitive interview study or other pilot test was not performed, only the first standard in part

1b needs to be completed and the rest of the box can be skipped. For rating the standards, the “worst score counts” method is used. A total rating for the box can be obtained

by taking the lowest rating of any standard in the box. It is also possible to obtain total ratings for different parts of the boxes by taking the lowest rating of any standard of that

part of the box.

### Box 1. PROM development

| **1a. PROM design** | |  |  |  |  |  |  |  |  |  |
| --- | --- | --- | --- | --- | --- | --- | --- | --- | --- | --- |
| *General design requirements* | | **Very Good** |  | **Adequate** |  | **Doubtful** |  | **Inadequate** |  | **Not applicable** |
| 1 | Is a clear description provided of the construct to be measured? | Construct clearly described |  |  |  |  |  |  |  |  |
|  |  |  |  |  |  |  |  |  |  |  |
|  |  |  |  |  |  |  |  |  |  |  |
|  |  |  |  |  |  |  |  |  |  |  |
| 2 | Is the origin of the construct clear: was a theory, conceptual framework or disease model used or clear rationale provided to define the construct to be measured? | Origin of the construct clear |  |  |  |  |  |  |  |  |
|  |  |  |  |  |  |  |  |  |  |  |
| 3 | Is a clear description provided of the target population for which the PROM was developed? | Target population clearly described |  |  |  |  |  |  |  |  |
|  |  |  |  |  |  |  |  |  |  |  |
|  |  |  |  |  |  |  |  |  |  |  |
|  |  |  |  |  |  |  |  |  |  |  |
| 4 | Is a clear description provided of the context of use | Context of use clearly described |  |  |  |  |  |  |  |  |
|  |  |  |  |  |  |  |  |  |  |  |
|  |  |  |  |  |  |  |  |  |  |  |

| *General design requirements* | | **Very Good** |  | **Adequate** |  | **Doubtful** |  | **Inadequate** |  | **Not applicable** |
| --- | --- | --- | --- | --- | --- | --- | --- | --- | --- | --- |
| 5 | Was the PROM development study performed in a sample representing the target population for which the PROM was developed? | Study performed in a sample representing the target population |  |  |  |  |  |  |  |  |
|  |  |  |  |  |  |  |  |  |  |  |
|  |  |  |  |  |  |  |  |  |  |  |
|  |  |  |  |  |  |  |  |  |  |  |
| *Concept elicitation (relevance and comprehensiveness)* | |  |  |  |  |  |  |  |  |  |
| 6 | Was an appropriate qualitative data collection method used to identify relevant items for a new PROM? |  |  | Assumable that the qualitative method was appropriate and suitable for the construct and study population, but not clearly described |  |  |  |  |  |  |
|  |  |  |  |  |  |  |  |  |  |  |
|  |  |  |  |  |  |  |  |  |  |  |
|  |  |  |  |  |  |  |  |  |  |  |
|  |  |  |  |  |  |  |  |  |  |  |
|  |  |  |  |  |  |  |  |  |  |  |
|  |  |  |  |  |  |  |  |  |  |  |
|  |  |  |  |  |  |  |  |  |  |  |
|  |  |  |  |  |  |  |  |  |  |  |
| 7 | Were skilled group moderators/interviewers used? |  |  |  |  |  |  |  |  | Not applicable |
|  |  |  |  |  |  |  |  |  |  |  |
|  |  |  |  |  |  |  |  |  |  |  |
| 8 | Were the group meetings or interviews based on an appropriate topic or interview guide? |  |  |  |  |  |  |  |  | Not applicable |
|  |  |  |  |  |  |  |  |  |  |  |

| *General design requirements* | | **Very Good** |  | **Adequate** |  | **Doubtful** |  | **Inadequate** |  | **Not applicable** |
| --- | --- | --- | --- | --- | --- | --- | --- | --- | --- | --- |
| 9 | Were the group meetings or interviews recorded and transcribed verbatim? |  |  |  |  |  |  |  |  | Not applicable |
|  |  |  |  |  |  |  |  |  |  |  |
| 10 | Was an appropriate approach used to analyse the data? |  |  |  |  |  |  |  |  | Not applicable |
|  |  |  |  |  |  |  |  |  |  |  |
| 11 | Was at least part of the data coded independently? |  |  |  |  |  |  |  |  | Not applicable |
|  |  |  |  |  |  |  |  |  |  |  |
| 12 | Was data collection continued until saturation was reached? |  |  |  |  |  |  |  |  | Not applicable |
|  |  |  |  |  |  |  |  |  |  |  |
| 13 | For quantitative studies (surveys): was the sample size appropriate? | ≥100 |  |  |  |  |  |  |  |  |

| **1b. Cognitive interview study or other pilot test** | |  |  |  |  |  |  |  |  |  |
| --- | --- | --- | --- | --- | --- | --- | --- | --- | --- | --- |
|  |  | **Very Good** |  | **Adequate** |  | **Doubtful** |  | **Inadequate** |  | **Not applicable** |
| 14 | Was a cognitive interview study or other pilot test conducted? | YES |  |  |  |  |  |  |  |  |
|  |  |  |  |  |  |  |  |  |  |  |
| *General design requirements* | |  |  |  |  |  |  |  |  |  |
| 15 | Was the cognitive interview study or other pilot test performed in a sample representing the target population? | Study performed in a sample representing the target population |  |  |  |  |  |  |  |  |
|  |  |  |  |  |  |  |  |  |  |  |
|  |  |  |  |  |  |  |  |  |  |  |
|  |  |  |  |  |  |  |  |  |  |  |
|  |  |  |  |  |  |  |  |  |  |  |
|  |  |  |  |  |  |  |  |  |  |  |
| *Comprehensibility* | |  |  |  |  |  |  |  |  |  |
| 16 | Were patients asked about the comprehensibility of the PROM? |  |  |  |  | Not clear **(SKIP standards 17-35)** |  |  |  |  |
|  |  |  |  |  |  |  |  |  |  |  |

## COSMIN box 2. Standards for evaluating the quality of content validity studies of PROMs

### Box 2. Content validity

Only those parts of the box need to be completed for which information is available. For example, if a content validity study was not performed in professionals, parts 2d and 2e do

not need to be completed. If patients were included in a content validity study, but they were only asked about comprehensibility of the PROM items, sections 2a and 2b do not need

to be completed.
For rating the standards, the “worst score counts” method is used. A total score for the box can be obtained by taking the lowest rating of any standard in the box. It is also possible to

obtain total ratings for different parts of the boxes by taking the lowest rating of any standard of that part of the box.

Content validity study was not performed in professionals or patients.
